# Residue-specific structures and membrane locations of pH-low insertion peptide by solid-state nuclear magnetic resonance

**DOI:** 10.1038/ncomms8787

**Published:** 2015-07-21

**Authors:** Nicolas S. Shu, Michael S. Chung, Lan Yao, Ming An, Wei Qiang

**Affiliations:** 1 Department of Chemistry, State University of New York, Binghamton, New York 13902, USA; 2 Department of Physics, Applied Physics and Astronomy, State University of New York, Binghamton, New York 13902, USA

## Abstract

The pH-low insertion peptide (pHLIP) binds to a membrane at pH 7.4 unstructured but folds across the bilayer as a transmembrane helix at pH∼6. Despite their promising applications as imaging probes and drug carriers that target cancer cells for cytoplasmic cargo delivery, the mechanism of pH modulation on pHLIP-membrane interactions has not been completely understood. Here, we show the first study on membrane-associated pHLIP using solid-state NMR spectroscopy. Data on residue-specific conformation and membrane location describe pHLIP in various surface-bound and membrane-inserted states at pH 7.4, 6.4 and 5.3. The critical membrane-adsorbed state is more complex than previously envisioned. At pH 6.4, for the major unstructured population, the peptide sinks deeper into the membrane in a state II′ that is distinct from the adsorbed state II observed at pH 7.4, which may enable pHLIP to sense slight change in acidity even before insertion.

## Abstract (web summary)

The pH-low insertion peptides (pHLIPs) respond to environmental pH variations by forming transmembrane α-helices. Here, the authors present the residue-specific structures and membrane locations of pHLIPs at different pH levels to probe the mechanism of their pH-dependant membrane insertion.

## Graphical abstract

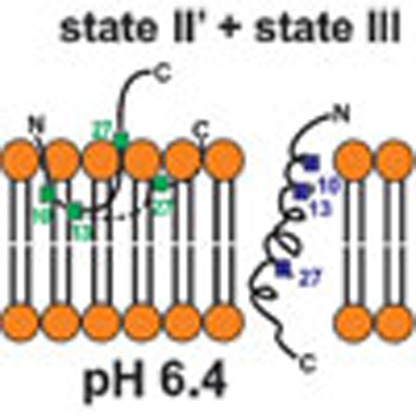

Localized extracellular acidity in solid tumours may be exploited for cancer diagnosis and treatment^1^,^2^,^3^,^4^. To this end, the 36-residue pH-low insertion peptide (pHLIP, with the sequence GGEQNPIYWARYADWLFTTPLLLLDLALLVDADEGT) has been developed as imaging tools and carriers of therapeutic agents^5^,^6^,^7^,^8^,^9^,^10^,^11^,^12^,^13^,^14^,^15^,^16^,^17^,^18^,^19^,^20^. The tumour-targeting ability of pHLIP is thought to be based on its insertion into membrane in response to environmental acidity^8^,^21^,^22^,^23^,^24^. Similarly, pHLIP can detect other pathological acidic microenvironments *in vivo*, such as those found in inflammation and ischaemic myocardium^8^,^25^,^26^. In addition, the transmembrane (TM) insertion behaviour endows pHLIP with a novel, built-in mechanism for cytoplasmic cargo delivery^5^,^22^,^27^,^28^,^29^,^30^,^31^,^32^,^33^. Molecules as varied as fluorescent dyes^17^,^22^, polar membrane-impermeable peptides (for example, phalloidin and other toxins)^29^,^30^,^33^,^34^, and chemotherapy drugs such as paclitaxel^27^ have been translocated and released into cells when attached to the inserting carboxy (C) terminus of pHLIP. Recently, pHLIP-mediated delivery of antisense PNA successfully silenced the miR-155 onco-miR in mouse lymphoma models^5^.

From a theoretical perspective, pHLIP is an important model system for the study of α-helix formation in the membrane environment, as well as α-helix insertion into the lipid bilayer^21^,^23^,^35^,^36^,^37^. Engelman, Reshetnyak, Andreev and co-workers established the pH-dependent folding and insertion behaviours of pHLIP using a variety of methods including circular dichroism (CD) and Trp fluorescence (both in kinetic stop-flow assays and under equilibrium conditions)^21^,^23^,^35^,^36^,^37^. In the pH range 6–8 and concentrations below 10 μM, pHLIP mainly exists as unstructured monomers in solution (that is, state I); At pH 7–8 and in the presence of lipid bilayers, pHLIP binds to the membrane but remains in the random coil conformation (that is, state II); Under slightly acidic conditions around pH 6, pHLIP folds into an TM α-helix (that is, state III)^21^,^23^. Two Asp residues, D14 and D25 in the TM region, were considered to be crucial for the pH modulation mechanism^38^. The protonation of their carboxylate side chains seem to trigger pHLIP insertion, as mutations at these sites can alter the pH_50_ (that is, the pH at which 50% of pHLIP peptides are inserted) and the cooperativity of insertion (that is, sharpness of pH-response)^27^,^39^, or abolish the pH-dependence of insertion altogether^8^,^9^,^40^. In a broader view, the mechanism of pHLIP insertion may share similarity with other peptides and proteins that insert into membrane in response to acidity, such as the GALA peptide^41^ and the translocation domain of A/B type bacterial toxins^42^.

Despite the potential biomedical applications and the theoretical importance of pHLIP-membrane insertion biophysics, the molecular details of the pH modulation mechanism of pHLIP-membrane interactions have not been thoroughly investigated. In particular, there has not been high-resolution structural data on the specific interactions between pHLIP and membrane lipids, which may shed light on detailed folding/insertion mechanisms. Due to the low-resolution nature of the previous methods (CD and Trp fluorescence), the critical membrane-embedded peripheral state II has been assumed to be homogeneous^23^. If pHLIP insertion can be viewed as a process involving interactions with lipids, protonation events and folding of the TM α-helix, high-resolution structural data can decipher each component in a pH- and residue-specific manner. Such detailed understanding of the mechanism of pH modulation on pHLIP insertion can facilitate the rational design of variants with improved pH response *in vivo*.

In this work, we describe the first nuclear magnetic resonance (NMR) study for pHLIP in 1-palmitoyl-2-oleoyl-*sn*-glycerol-3-phosphocholine (POPC) membranes at pH 7.4, 6.4 and 5.3. Solid-state NMR (ssNMR) spectroscopy is especially suitable for probing the structures and lipid interactions of membrane-associated peptides and proteins, because of the non-crystalline and insoluble nature of the samples^43^. By incorporating isotope-labelled amino acids at different sites along the sequence, residue-specific information about pHLIP structures and membrane locations are obtained at these different pH values. Our results reveal a more complex picture of the membrane-adsorbed state of pHLIP than previous perceived. In addition, the study provides direct evidence for the co-existence of both peripheral and inserted pHLIP molecules at an intermediate pH of 6.4.

## Results

### Validation of NMR sample preparation protocol

**Figure 1:**
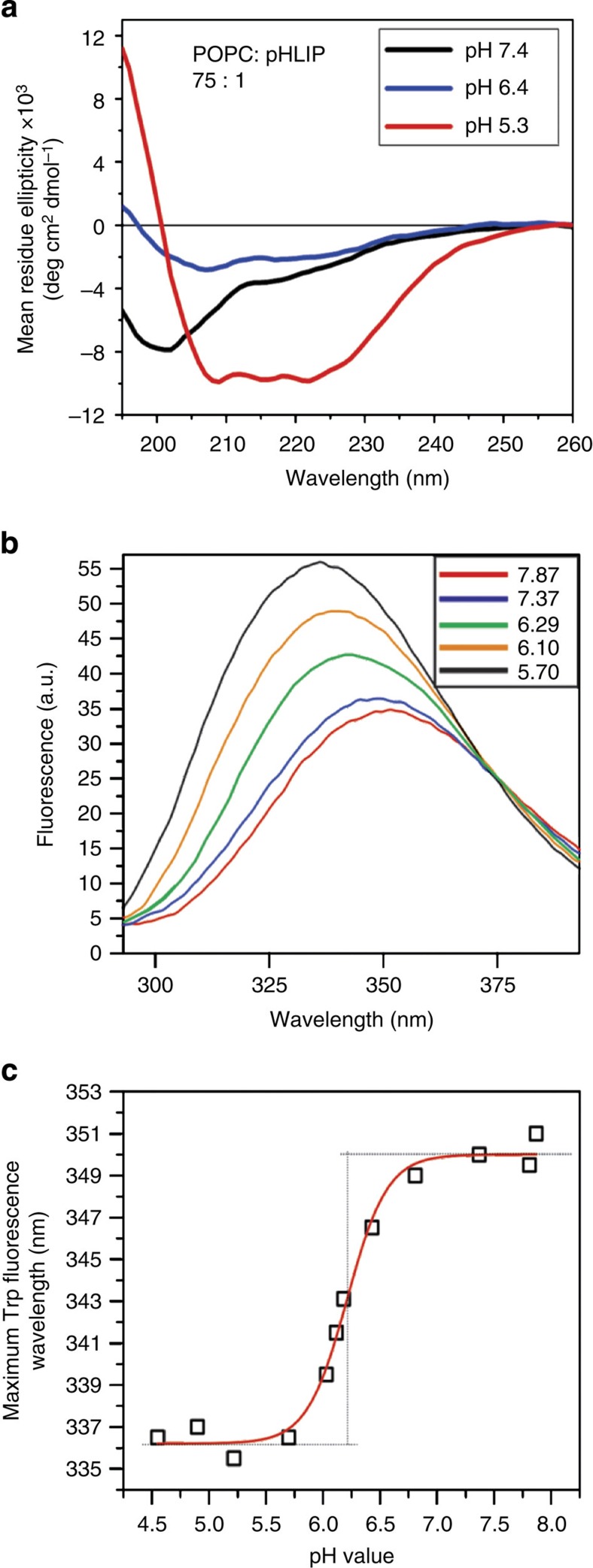
Biophysical characterizations of the NMR samples. (**a**) CD spectra for NMR samples with 1:75 P/L ratio prepared at pH 7.4 (black), pH 6.4 (blue) and pH 5.3 (red). (**b**) Representative tryptophan fluorescence spectra at different pH values. The spectra were colour coded as shown in the inset. For each individual pH, fluorescence spectra were recorded three times. (**c**) Plot of maximum wavelengths in tryptophan fluorescence spectra as a function of sample pH. The data were fit to a typical pHLIP transition curve with pH_50_=6.21±0.06.

Due to the low sensitivity of ssNMR, samples need to be prepared with a relatively high peptide-to-lipid ratio (P/L). The 1:75 P/L ratio was chosen because (a) it provided reasonable signal for ssNMR experiments and (b) pHLIP peptides exist as monomers at this ratio in both states II and III^23^. Samples were prepared by adding pHLIP to POPC liposomes in two sequential steps (1:150 P/L ratio in each step, final [pHLIP] ∼10 μM). Figure 1 shows the CD and Trp fluorescence measurements on the final samples prepared using this approach. The CD spectra in Fig. 1a indicate a conformational change from random coil to α-helix as the pH decreased from 7.4 to 5.3, which are consistent with previous observations^21^,^23^. At pH 6.4, the CD spectrum contains features of α-helix with minima at ∼208 and ∼220 nm; however, the molar ellipticities are only ∼30% of those observed at pH 5.3. One possibility is that the sample at pH 6.4 is heterogeneous, containing a small population of helical pHLIP and a larger portion of non-helical peptides. Alternatively, it is possible that a lower fraction of pHLIP sequence adopts helical conformation at this intermediate pH. Our NMR data (to be discussed below) supports the former notion. Furthermore, samples prepared with a larger 1:300 P/L ratio gave similar CD spectra at pH 7.4, 6.4 and 5.3 (Supplementary Fig. 1) to those obtained with the 1:75 P/L ratio.

To further validate the biophysical relevance of our NMR samples, Trp fluorescence was measured for pHLIP/POPC samples prepared using the step-wise addition protocol. As shown in Fig. 1b,c, titration from pH 8 to 4 induced an increase in Trp emission intensity accompanied by a blue shift in emission *λ*_max_. Judging from the pH versus emission *λ*_max_ plot (Fig. 1c), the transition pH_50_ is ∼6.2. Both of these observations agree well with literature precedents and reflect the change of membrane locations (that is, deeper in the membrane at pH 4 than at pH 8) for pHLIP Trp residues W9 and W15 upon insertion^21^,^23^. These CD and Trp fluorescence data confirmed that the step-wise sample preparation protocol generated biophysically well-behaved pHLIP/POPC systems, which were used in ssNMR experiments after centrifugation.

### Restriction of lipid head group motion upon pHLIP insertion

**Figure 2:**
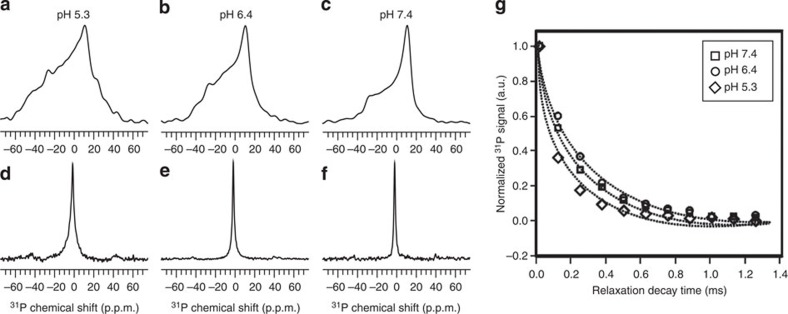
^31^P solid-state NMR spectroscopy. (**a**–**c**) Static ^31^P spectra for samples prepared at different pH values. All the spectra were processed with 100 Hz Gaussian line broadening. (**d**–**f**) The MAS ^31^P spectra for samples at different pH. There was no additional line broadening during the spectral processing. All the spectra were referenced to 80% H_3_PO_4_ at 0 p.p.m. (**g**) Plot of ^31^P T_2_ relaxation decay curves for pH 7.4 (squares), 6.4 (circles) and 5.3 (diamonds). The dashed lines show the data fitting to a single exponential decay.

The ^31^P static and magic angle spinning (MAS) ssNMR spectra were obtained for pHLIP/POPC samples prepared at pH 7.4, 6.4 and 5.3 (Fig. 2). At pH 7.4 and 5.3, pHLIP should exist in states II and III respectively. Figure 2a–c illustrate variations in ^31^P powder pattern due to pH change: Samples at pH 7.4 showed a typical ^31^P powder pattern for a lamellar liquid crystalline (*L*α) phase membrane, with a chemical shift anisotropy parameter of ∼60 p.p.m. and an asymmetry parameter of 0 (ref. 44). For pH 6.4 and 5.3 samples, the chemical shift anisotropy increased to 75 and 105 p.p.m. while the asymmetry parameters changed to 0.42 and 0.33, respectively. The non-axial symmetric features in pH 6.4 and 5.3 samples indicate different ^31^P chemical shielding along the three dimensions, which suggest that the lipid head groups may have less free rotational motion at pH 5.3 and 6.4 than at pH 7.4.

The MAS ^31^P spectra (Fig. 2d–f) show single peaks at the same chemical shift for all the pH values, which confirmed that pHLIP insertion does not disrupt the overall lipid bilayer structure. However, broadenings of peaks at pH 5.3 and 6.4 versus 7.4 again suggest restricted motion for the ^31^P nuclei and stronger dipolar coupling interactions at lower pH values.

To further investigate membrane perturbation, the ^31^P spin–spin relaxation time constants (T_2_) were measured. Figure 2g plots the exponential decay curves at different pH values, which are distinguishable within experimental errors. The decay rate was clearly faster at pH 5.3 than at pH 6.4 or 7.4. The best-fit T2 constants for pH 7.4, 6.4 and 5.3 samples are 1.23±0.03 ms, 1.28±0.02 ms and 0.92±0.03 ms, respectively. Since a longer T_2_ time constant indicates higher mobility of ^31^P-containing lipid head groups^45^, these results further suggest that the sample at pH 5.3 (that is, with pHLIP in state III) contains POPC lipids with reduced dynamics.

### Residue-specific secondary structures at different pH values

**Figure 3:**
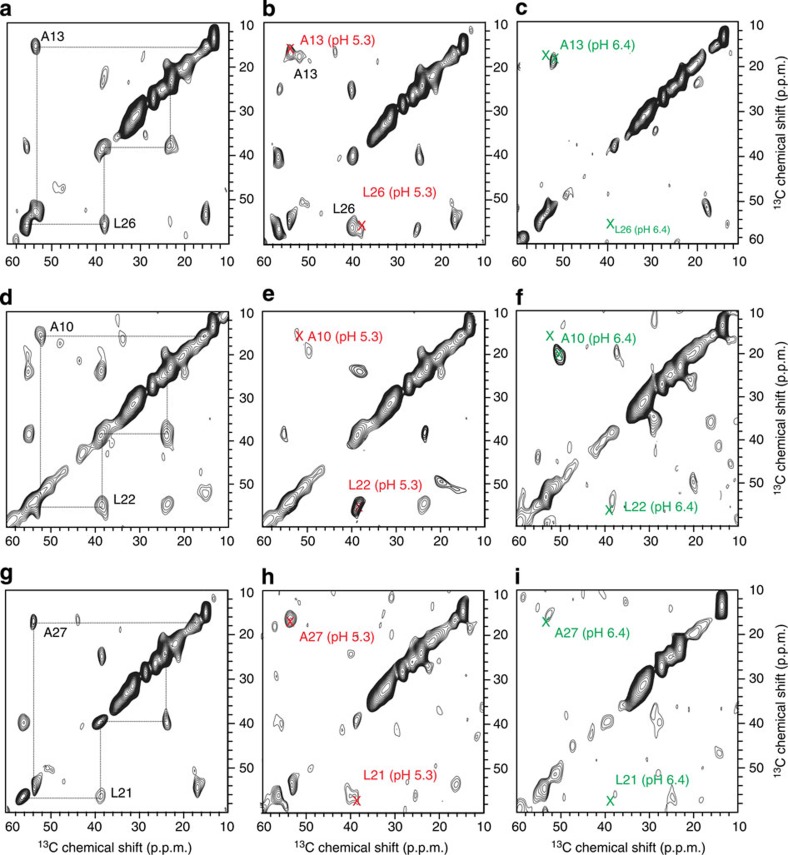
The aliphatic regions of the 2D ^13^C–^13^C spin diffusion spectra. (**a**) A13, L26 labelled, pH 5.3, (**b**) A13, L26 labelled, pH 6.4, (**c**) A13, L26 labelled, pH 7.4, (**d**) A10, L22 labelled, pH 5.3, (**e**) A10, L22 labelled, pH 6.4, (**f**) A10, L22 labelled, pH 7.4, (**g**) L21, A27 labelled, pH 5.3, (**h**) L21, A27 labelled, pH 6.4, (**i**) L21, A27 labelled, pH 7.4. In **a**, **d** and **g**, dashed lines were utilized to highlight the intramolecular cross peaks used for chemical shift analysis. Coloured crosses were used to highlight the peak shifts at different pH values. All the spectra were processed with 100 Hz Gaussian line broadening in both dimensions, and were referenced to ^13^C′ of a standard alanine sample at 177.95 p.p.m.

**Figure 4:**
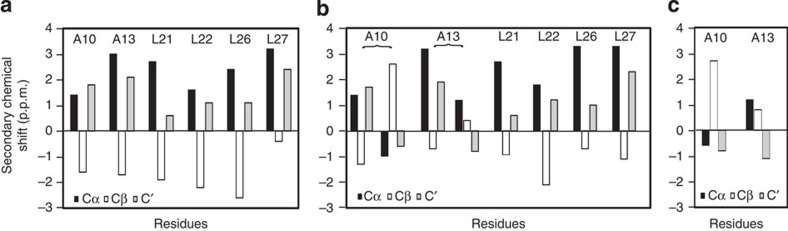
Plots of the secondary chemical shifts for Cα, Cβ and C'. (**a**) pH 5.3, (**b**) pH 6.4, (**c**) pH 7.4. Panel **a** indicates a strongly α-helical conformation in state III. In **b**, a mixture of helical state III and non-structured state II′ is observed. The parentheses indicate multiple conformations within one residue. In **c**, only A10 and A13 showed non-helical structures at pH 7.4 (state II).

We chemically synthesized pHLIP peptides with ^13^C uniform isotopic labelling at different Ala/Leu pairs A13/L26, A10/L22 and A27/L21. These labelled residues are located within the proposed TM helix segment of state III pHLIP^21^. The aliphatic region of the two-dimensional (2D) ^13^C–^13^C spin diffusion spectra are shown in Fig. 3 and the chemical shift values are summarized in Table 1. Plots of secondary chemical shifts (Δ*δ*) of Cα, Cβ and C′, obtained by subtracting Ala/Leu random coil reference chemical shift values^46^ from the observed chemical shift values, are shown in Fig. 4.

At pH 5.3, the secondary chemical shifts of all the labelled residues (Fig. 4a) showed the typical pattern for α-helix, with positive, negative and positive deviations for Cα, Cβ and C′ respectively^46^. This is in agreement with previous proposal that all these residues are located in the TM helical region of inserted pHLIP in state III^21^. Spectra at pH 7.4 generally showed weaker and fewer cross-peaks than those at pH 5.3 (Fig. 3). The Cα/Cβ cross-peaks were only observed for A10 and A13, but not for L21, L22, L26 and A27 (Fig. 3c,f,i), indicating that at pH 7.4 the C-terminal part of the TM sequence does not have a well-defined secondary structure and is more flexible than the amino (N)-terminal region. The secondary chemical shifts of A10 at pH 7.4 showed typical β-strand (that is, negative/positive/negative deviations for Cα/Cβ/C′ respectively) and A13 did not show clear pattern for regular secondary structures (Fig. 4c). This observation is consistent with the CD data that pHLIP in state II at pH 7.4 is largely random coil^21^,^23^.

The overall secondary chemical shift patterns at pH 6.4 resemble a combination of those observed at pH 7.4 and 5.3 (Fig. 4). They show two populations that differ in secondary structure for A10 and A13, while L21, L22, L26 and A27 have only helical signatures (Fig. 4b). The Cα/Cβ cross-peaks at pH 6.4 also seem to be a composite of spectra from pH 5.3 and 7.4 (Fig. 3 and Table 1). For most residues (except L21 and L26), the Cα/Cβ cross-peaks of the helical population at pH 6.4 overlap well with cross-peaks at pH 5.3, while the second set of cross-peaks for A10 and A13 overlap with those at pH 7.4. For L21 and L26, the overlap with pH 5.3 cross-peaks appear to be on the shoulders of dominant peaks that are distinct, which could mean that the inserted state III at pH 6.4 may be slightly different from that at pH 5.3. In general, the signal intensities of cross-peaks of the helical population at pH 6.4 are much less than those at pH 5.3, suggesting that it is a minor population at pH 6.4. Together, these NMR data provide direct evidence that pHLIP/POPC at pH 6.4 exist as a mixture of a largely unstructured population (state II′) and an inserted α-helical population (state III).

### Residue-specific membrane locations at different pH values

**Figure 5:**
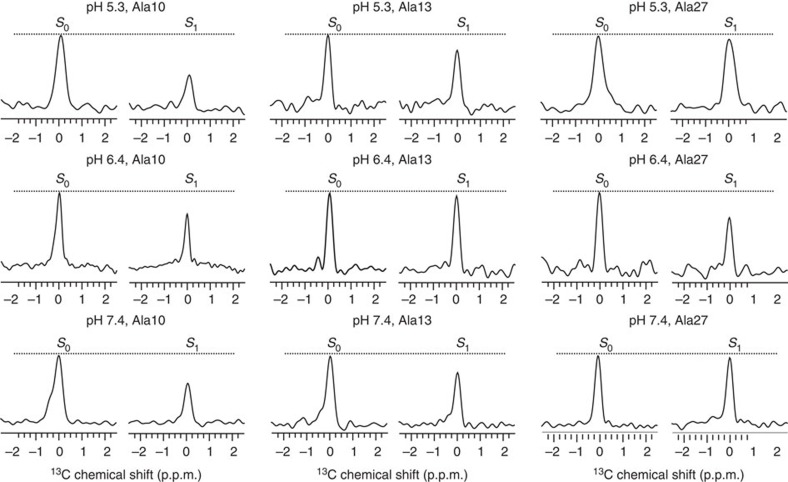
Representative ^13^C–^31^P *fs*REDOR spectra taken at 17.8 ms dephasing time. The dashed lines on top of each spectra pair (*S*_0_ and *S*_1_) highlight the peak intensities in *S*_0_. All the spectra were processed with 10 Hz Gaussian line broadening. Each NMR spectrum was collected with ∼40 k scans (that is, ∼24 h).

**Figure 6:**
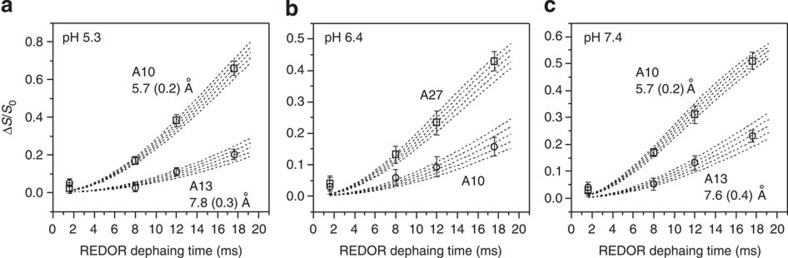
Experimental and simulated ^13^C–^31^P REDOR dephasing. (**a**) pH 5.3, (**b**) pH 6.4, (**c**) pH 7.4. Experimental data were presented as open symbols with s.d. that were determined from the spectral noise. The dashed lines represented simulated REDOR dephasing curves within the deviation *χ*=*χ*_min_±1 (approximately with the ^13^C–^31^P dipolar coupling frequency range *f*=*f*_opt_±4 Hz, where *f*_opt_ is the best-fit frequency) to provide a qualitative view of the best-fit distances. The best-fit distances were provided in the plots with uncertainties in parentheses.

The ^13^C–^31^P frequency-selective rotational-echo double-resonance (*fs*REDOR) NMR^47^ spectroscopy was used to quantify the membrane locations of labelled amino acids. The *fs*REDOR pulse sequence is developed to provide specific observation of a certain spectral region, which is particularly useful for our samples as the ^13^C signals are from both labelled amino acids and naturally abundant lipids. The ^31^P nuclei naturally exist at the membrane/water interface in the phosphate diester moieties of POPC lipid head groups. We selected the Ala ^13^Cβ signals for REDOR analysis because they do not overlap with lipids or Leu ^13^C signals. The Gaussian selective pulse was set to 6.0 ms to provide a ±300 Hz (that is, ∼4.0 p.p.m. in ^13^C spectra) observation window, according to previous studies^47^. This observation range covered the chemical shifts for Ala Cβ based on our 2D spin diffusion experiments (Table 1). The pulsed-spin locking (PSL) technique^48^ was applied during the acquisition time to increase the signal-to-noise ratio thus facilitating quantification. The representative REDOR full (*S*_0_) and reduced (*S*_1_) ^13^C spectra with the longest dephasing time (that is, 17.8 ms) are shown in Fig. 5, and the plots of dephasing (Δ*S/S*_0_) as a function of dipolar evolution time are provided in Fig. 6, along with the theoretical fitting curves. A detailed description of the calculation of Δ*S/S*_0_ is provided in the Supplementary Methods.

Owing to the utilization of PSL, for an individual sample, the REDOR spectra do not contain chemical shift information. If the sample contained multiple structures (as in the case of the pH 6.4 sample), different conformations at the labelled site will all contribute to the REDOR spectra. Thus, information about a major population can only be obtained by removing the contribution of the minor population from the overall signal. Our data fitting was based on the following assumptions: (a) The pH 5.3 sample only contained TM inserted pHLIP in state III, (b) the pH 7.4 sample only contained membrane-adsorbed pHLIP in state II and (c) the pH 6.4 sample contained a mixture of inserted state III (∼30%) and adsorbed state II′ (∼70%). This model is based on our 2D ^13^C NMR, CD and Trp fluorescence results, as well as literature precedents^21^,^23^,^39^. For fitting at pH 6.4, we assumed that the 30% state III population has the same ^13^C–^31^P distances as the state III pHLIP at pH 5.3. By removing the contributions from this minor population, the best-fit distances discussed below for the pH 6.4 sample solely reflect the 70% state II′ major population.

The theoretical ^13^C–^31^P REDOR dephasing curves shown in Fig. 6 were generated using model systems that mimic the geometries in the surface-bound and membrane-inserted scenarios. For samples at pH 7.4 and 6.4 (that is, surface-bound states II/II′), the experimental data were fit to a four-spin system consisted of one ^13^C and three ^31^P nuclei, with a single fitting parameter to represent the distance between ^13^C and the phosphate head group plane defined by the three ^31^P nuclei. For samples at pH 5.3 (that is, inserted state III), the data were fit to a three-spin system made of one ^13^C and two ^31^P, because it seems that only two annular lipids may be in close proximity to a specific ^13^C nuclear in a given system. In this calculation, the distances are from the ^13^C to the centre of the imaginary line that connects the two ^31^P nuclei. More details about the geometries of the fitting models and the calculation for the theoretical curves are provided in Supplementary Fig. 2 and the Supplementary Methods.

At pH 7.4 (Fig. 6c), the β carbons of A10 and A13 were located 5.7±0.2 and 7.6±0.4 Å away from the ^31^P head groups, respectively. The Cβ of A27 did not show detectable REDOR dephasing within the experimental time, which indicated a longer ^13^C–^31^P distance (that is, probably >10.0 Å based on simulations). At pH 6.4 (Fig. 6b), the β carbons of A10 and A27 of the major membrane-adsorbed population showed close proximity to the ^31^P head groups, with the best-fit distances of 8.4±0.6 and 6.4±0.2 Å, respectively. In this case A13 was located more than 10.0 Å away from the head group ^31^P. Such results clearly indicate that the membrane-adsorbed state of pHLIP at pH 7.4 (state II) is different from the membrane-embedded state of pHLIP at pH 6.4 (state II′). At pH 5.3, the β carbons of A10 and A13 were located 5.7±0.2 and 7.8±0.3 Å away from the ^31^P nuclei, while A27 did not show obvious experimental decay (that is, >10.0 Å away). Thus, pHLIP in state III at pH 5.3 showed clear residue-specific differences in terms of both secondary structure and membrane location to the peripheral embedded state II′ at pH 6.4.

To provide additional information about the membrane location of A13, we performed ^13^C–^2^H REDOR experiments with singly ^13^C-labelled pHLIP at A13. For the membrane component, 15% of the phospholipids have fully deuterated alkyl chains. Samples prepared at all the pH values showed ∼30% REDOR dephasing in a 20 ms dipolar coupling evolution period (Supplementary Fig. 3), which is comparable to previously reported measurements on model TM and fusion peptides^49^. Qualitatively, the ^13^C–^2^H REDOR data suggest that A13 is in close proximity with membrane lipid fatty acid tails at all the pH values. Taken together with its distances to phosphate lipid head groups being 7.6 and >10 Å at pH 7.4 and 6.4, respectively, we arrived at the conclusion that A13 sinks deeper into the hydrophobic membrane interior as pH decreased from 7.4 to 6.4 in the pre-insertion state II to state II′ transition.

## Discussion

The combination of 2D spin diffusion and REDOR NMR spectroscopy provided a first glance at residue-specific structures and membrane locations of pHLIP at different pH values. The pH 7.4 and 5.3 conditions used are within the high and low plateau regions of the pHLIP Trp fluorescence blue-shift curve (Fig. 1b), which represent the well-described peripheral state II and membrane-inserted states III in previous studies, respectively^21^,^23^,^38^. A key value of this study is the characterization of membrane-associated pHLIP at the intermediate pH of 6.4, which is within the sharp transition region in the pH versus *λ*_max_ plot (Fig. 1b), but still higher than the pH_50_ value of 6.2.

At pH 7.4, the 2D ^13^C–^13^C spectra showed strong cross-peaks for the N-terminal residues A10 and A13, but not for the C-terminal residues L21, L22, L26 and A27 (Fig. 3). The random motion of A10 and A13 are restricted likely due to membrane association. On the contrary, the C-terminal residues seem to have a high degree of dynamics. Thus, these results suggest that it is the N-terminal segment around A10 and A13 that dominates the membrane binding process. The ^13^C–^31^P *fs*REDOR data (Figs 5 and 6), which revealed membrane locations of specific ^13^C-labelled residues, further support the N-terminal binding hypothesis. At pH 7.4, the ^13^Cβ of A10 and A13 were found to be 5.7±0.2 and 7.6±0.4 Å away from the plane defined by ^31^P nuclei, which is approximately located at the centre level of the PC head group layer that is ∼12 Å thick at the membrane-aqueous interface^46^. These distances would suggest that A10 and A13 are bound to the lipid head groups closely, perhaps anchored by R11 side chain guanidinium–phosphate interactions. In comparison, no ^13^C–^31^P *fs*REDOR dephasing was observed for A27, suggesting that this residue is not close to the ^31^P head group (>10 Å) at pH 7.4.

**Figure 7:**
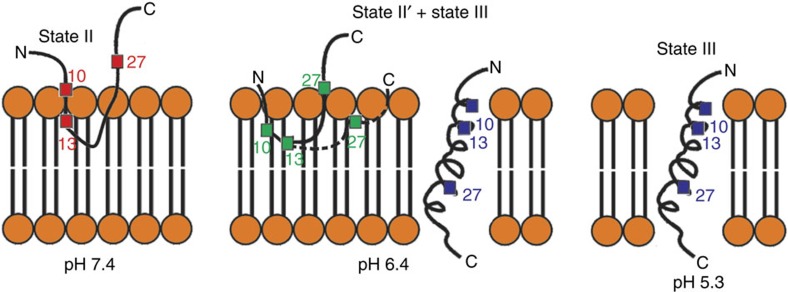
Cartoon models of the membrane locations of pHLIP. In state II (pH 7.4), the N-terminal A10 (5.7 Å) and A13 (7.6 Å) residues are in close proximity to the head group phosphates. The C-terminal A27 (>10 Å) is depicted as outside the membrane. In the adsorbed state II′ at pH 6.4, pHLIP sinks deeper into the bilayer (with A10 at 7.6 Å and A13 at >10 Å), pulling A27 (6.4 Å) into close distance to head group phosphates. In state III (at both pH 6.4 and 5.3), A10 (5.7 Å) and A13 (7.8 Å) are located deeper in the membrane than in state II (pH 7.4) but maintain similarly close distances to phosphates, whereas A27 is still >10 Å away from head group phosphates of the opposing monolayer lipids.

Furthermore, ^13^C–^2^H REDOR experiments with deuterated lipids showed that at pH 7.4 the Cβ of A13 is in close contact with the alkyl chains of fatty acids (Supplementary Fig. 3). Thus, in state II, A13 is located beneath the head group region in the membrane, as depicted in Fig. 7. This placement of A13 being 7.6±0.4 Å down from head group phosphates agrees well with previous Trp (W9/W15) fluorescence quenching data obtained using C6–7 or C9–10 dibromo PC lipids (sn-1 16:0, sn-2 18:0; ref. 50), by which Zoonens *et al*.^50^ defined the depth of W15 to be 9.7 Å from head group phosphates and 8 Å from DMPC bilayer centre at pH 7.4 (35 °C, 1:400 P/L ratio).

The ^13^C–^31^P *fs*REDOR data also showed that at pH 7.4 residue A27 is >10 Å away from the ^31^P nuclei. The model in Fig. 7 proposes that this residue is outside the membrane because of the highly negatively charged C terminus at pH 7.4 (that is, D25, D31, D33, E34 and C-terminal carboxylates). The membrane location for the linker region between A13 and A27 in state II as depicted in Fig. 7 is putative, based on presumed favourable partitioning of the hydrophobic sequence of WLFTTPLLLL (residues 15–24) into the membrane. We emphasize that this model may only represent pH 7.4 state II under the ‘crowded' conditions (that is, P/L at 1:75; ref. 36). It remains to be seen whether A27 is closer to the head group phosphates under less crowded conditions.

The ^13^C 2D NMR spectra for the pH 5.3 sample show a single set of cross-peaks with strong intensities for all the labelled sites (Fig. 3), which suggest a well-ordered structure. The secondary chemical shift patterns confirmed the dominant α-helix conformation between residues A10 and A27 (Fig. 4a). Extensive biophysical studies have shown that pHLIP forms a TM helix (that is, state III) at pH 5.3 (refs 21, 35). Our 2D NMR data further support this conclusion. Accordingly, the ^13^C–^31^P *fs*REDOR data should provide residue-specific membrane locations of the inserted TM helix. At pH 5.3, the ^13^Cβ of A10, A13 or A27 is located 5.7±0.2, 7.8±0.3 or >10 Å away from the closest pair of ^31^P nuclei, respectively. Considering the presence of phosphate oxygen atoms (which have a Van der Waals radius of 1.5 Å and a P–O bond length of 1.5 Å) and Ala methyl hydrogen atoms (which have a Van der Waals radius of 1.2 Å and a C–H bond length of 1.1 Å), a ^13^Cβ–^31^P internuclear distance of 5.7±0.2 Å would suggest that the A10 ^13^Cβ must be near level with the head group phosphates. Additional ^13^C–^2^H REDOR with deuterated lipids showed that at pH 5.3 the Cβ of A13 is in close contact with lipid alkyl chains (Supplementary Fig. 3). Thus, A13 is more deeply inserted in the membrane than A10, which supports the notion that pHLIP insertion is unidirectional with C terminus translocated across the membrane^22^,^28^. On the basis of such an insertion geometry, A27 would be ∼26 Å away from the ^31^P nuclei of the top lipid monolayer and thus ∼13 Å away from the head group phosphate plane on the other side of the POPC membrane bilayer. Experimentally, no ^13^C–^31^P REDOR dephasing was observed from A27, which is consistent with the described model.

Chemical shift analysis of ^13^C 2D NMR indicates that there are at least two populations of structures at pH 6.4 (Figs 3 and 4b)—a major unstructured population (that is, state II′) and a minor helical population (similar to state III observed at pH 5.3). As CD and Trp fluorescence data (Fig. 1) suggest that ∼30% of pHLIP peptides are helical and inserted at pH 6.4, contribution from this minor state III population to the pH 6.4 ^13^C–^31^P *fs*REDOR data was removed during fitting. Thus, solely regarding the membrane-embedded state II′ population, the best-fit distances between ^13^Cβ and ^31^P for residues A10, A13 and A27 are 8.4±0.6, >10 and 6.4±0.2 Å. These state II′ values differ considerably from those of the adsorbed state II at pH 7.4, in which A10 (5.7±0.2 Å) and A13 (7.6±0.4 Å) are in closer proximity to the phosphate while A27 (>10 Å) is further away. Additional ^13^C–^2^H REDOR data obtained using deuterated lipids further suggest that at pH 6.4, the Cβ of A13 has close contact with the lipid alkyl chains (Supplementary Fig. 3). Thus, A13 is buried in the membrane at a depth of >10 Å from the head group phosphate groups. We propose that in response to the slight acidity change from pH 7.4 to 6.4, the N-terminal region of pHLIP sinks deeper into the hydrophobic interiors of the lipid bilayer, thus dragging A27 and the polar C terminus to close proximity of the head group phosphates.

It has been proposed that the pH modulation of membrane insertion of pHLIP is initiated by the protonation of D14/D25 side chains^8^,^21^,^38^,^39^,^40^. The pKa value of the side chain carboxyl group of Asp in aqueous solution is ∼4.0, which is lower than the range of pH variation in the present study (that is, 5.3–7.4). However, the actual pKa of Asp side chains may be sensitive to the local environment. The protonated state can be stabilized by specific local interactions. For example, it was previously reported that the pKa of the side chain of Asp26 in *E. coli* thioredoxin is shifted to 7.3–7.5 due to the formation of a water-mediated hydrogen bond^51^. A more nonpolar environment, that is, the one with lower dielectric constant than water (such as the membrane interiors), should also favour the formation of the neutral carboxylic acid, effectively increasing the pKa of D14/D25 in membrane-bound states. Considering the locations of A13 in state II/II′ (that is, 7.6 Å/>10 Å down from head group phosphates), the neighbouring D14 side chain should also be among the hydrophobic alkyl chains, and thus may already be protonated at pH 6.4–7.4.

The linker region between A13 and A27 is long and hydrophobic, with the sequence WLFTTPLLLLDL between residues 15 and 26. During state II to state II′ transition, to have such a dramatic pulling effect on A27 (from >10 Å outside the membrane in state II to 6.4±0.2 Å in state II′), the D25 side chain may also become kinetically protonatable at pH 6.4. We do not know whether A27 is located 6.4 Å above or below the phosphate groups, and therefore, both models are presented in Fig. 7. Once D25 is protonated, the LALLV linker between residues 26 and 30 may in turn pull D31, D33 and E34 of the C terminus into membrane environments for protonation, ultimately reaching the equilibrium ratio of ∼30% inserted (that is, state III) : 70% embedded (that is, state II′) at pH 6.4. In this proposed model, the protonation of D25 is the master switch that controls insertion, which is consistent with the fact that small modifications in D25 side chain can significantly alter the pH_50_ of insertion^27^,^39^. The interlacing of polar but neutralizable Asp residues and hydrophobic sinker stretches (that is, WLFTTPLLLL between D14 and D25 and LALLV between D25 and D31) may allow pHLIP to respond to slight change in acidity with impressive cooperativity. Future NMR characterization will be performed to study the pH-dependent protonation states of these key Asp residues. Last, these data also raise the possibility that pHLIP may respond to slight acidity in cellular and *in vivo* experiments through the state II to state II′ transition, with the latter intermediate state II′ supporting facile insertion by anchoring the C-terminal region surrounding D25 more deeply in the membrane.

## Methods

### Peptide synthesis and purification

All the isotope-labelled pHLIP (NH_2_-GGEQNPIYW**A**RY**A**DWLFTTP**LL**LLD**LA**LLVDADEGT-CO_2_H, with labelled sites shown in bold) were synthesized manually using the routine 9-fluorenylmethyloxycarbonyl (Fmoc) protecting group chemistry with preloaded Fmoc-Thr(*t*Bu)-Wang resin (0.3 mmol g^−1^ substitution, AAPPTec Inc., Louisville, KY). The isotope-labelled amino acids were purchased from Cambridge Isotope Laboratory, Inc. (Tewksbury, MA), and Fmoc protection of the free amino group was accomplished using literature approaches^52^. The crude peptides were purified using high-performance liquid chromatography (Agilent HP 1100 series, Agilent Technologies Inc., Santa Clare, CA) installed with a Zorbax C18 reversed-phase column (Agilent Technologies Inc., Santa Clare, CA). A linear gradient with water and acetonitrile was utilized for the purification. The final products were verified with matrix-assisted laser desorption/ionization–time of flight mass spectrometry (Mass Spectrometer Facility, University of Illinois).

### Preparation of pHLIP/POPC liposome NMR samples

All the samples had the final pHLIP concentration ∼10 μM and P/L ratio of ∼1:75. It was previously shown that the peptides exist mainly as monomers under these conditions^23^. The sample preparation started from evaporating the chloroform from the 1-palmitoyl-2-oleoyl-*sn*-glycerol-3-phosphocholine (POPC, purchased from Avanti Polar Lipids, Inc. Alabaster, AL) lipids using N_2_ flow, and followed by high-vacuum drying for at least 8 h. The dried lipid film was then re-suspended thoroughly in the desired amount of phosphate buffer (1 mM, pH 8.0) based on the calculation of final peptide concentration. The liposome solution was then extruded using a 200 nm pore-size polycarbonate membrane (Avanti Polar Lipids, Inc. Alabaster, AL). The incubation process consisted of two additions of equal amount of pHLIP with the peptide-to-lipid ratio of 1:150 for each time. The peptide was initially dissolved in dimethyl sulfoxide (DMSO) to a concentration of ∼2 mM and then diluted into the liposome solution. After the first addition (initial overall P/L ratio of 1:150), the pHLIP/liposome sample was incubated at 4 °C for 24 h with gentle agitation on an incubator shaker (Boekel Industrial Inc., Feasterville, PA) before the second addition (which gave the final overall P/L ratio of ∼1:75). The pHLIP/POPC solution was incubated for another 48 h after the second addition. The pH of solution was checked during each peptide addition step and re-adjusted to 8.0 if necessary. The final pH values were adjusted to 5.3, 6.4 and 7.4 after the completion of the second incubation period, and then the samples were incubated quiescently for 1 h at ambient temperature before ultracentrifugation. NMR samples were collected by ultracentrifugation at 432,000*g* for 1 h at 4 °C. The gel-like wet pellet was packed into a solid-state NMR rotor using an Eppendorf centrifuge. The 280 nm absorbance of the supernatant was measured to ensure that >90% of pHLIP peptides are with the POPC liposomes.

### Tryptophan fluorescence assays

For Trp fluorescence experiments, the pHLIP/POPC solutions prepared as described above were adjusted to a series of samples with 20 different pH values using concentrated buffer solutions (containing 50 mM of sodium phosphate and 50 mM of sodium acetate). The final pH values ranged from 8.12 to 4.00. The actual pH values after the adjustment was determined right before the fluorescence measurements. Each solution sample at different pH values had an associated control sample that was prepared from the liposomes without pHLIP. All fluorescence spectra were recorded on a PerkinElmer LS55 fluorescence spectrometer (PerkinElmer Inc., Waltham, MA). The samples were excited at 285 nm with 10 nm slit width and the emission spectra were collected from 301 to 400 nm with 2.5 nm slit width and 300 nm min^−1^ scanning rate. For each sample, the associated background signal was subtracted from the raw data. The maximum peak wavelength was determined using the FL WinLab software associated with the spectrometer.

### CD spectroscopy

A lyophilized sample of pHLIP was dissolved in aqueous sodium phosphate (NaPi) buffer (1 mM, pH 8.2) to create a 10 μM stock solution (confirmed by ultraviolet absorption at 280 nm). The pH of this pHLIP stock solution was re-adjusted back to ∼8.1. This pHLIP stock solution was mixed with 50 nm POPC liposomes (smaller vesicles than those for NMR were used in CD experiments to reduce light scattering), and then further diluted with aqueous NaPi buffer (1 mM, pH 8.2) to give the following final concentrations: sample A (with P/L ratio of 1:150): [pHLIP] ∼5 μM, [POPC] ∼750 μM; sample B (with P/L ratio of 1:300): [pHLIP] ∼5 μM, [POPC] ∼1.5 mM. Equivalent samples without pHLIP (for background subtractions) were also prepared. These samples were incubated overnight (for at least 12 h) at 4 °C (sample B) or room temperature (sample A) to allow pHLIP to partition to the membrane surface. Following the step-wise sample preparation protocols for NMR, an additional equal volume of 5 μM pHLIP solution was added to sample A on the next day to reach P/L ratio of 1:75. Then, sample A and its corresponding control solution were incubated at room temperature for another 24 h. These solutions were used for CD measurements after adjusting the final pH values to 5.3, 6.4 and 7.4, and then allowed to incubate quiescently for 1 h at room temperature.

All the CD spectra were collected on a JASCO J-810 spectropolarimeter (Easton, MD) at 20 °C using a 1-mm cuvette (sample volume: 350 μl). Other detailed settings are as follows: sensitivity: 100 mdeg; start: 260 nm; end: 190 nm; data pitch: 1 nm; scanning mode: continuous; scanning speed: 50 nm min^−1^; response: 2 s; bandwidth: 2 nm; accumulation: 40 (each sample was measured 40 times and averaged). The raw data were converted to mean residue ellipticity (|*θ*|) using equation (1) below:


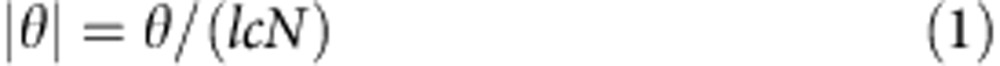


where *θ* represents the measured ellipticity (in the unit of millidegree), *l* represents path length (1 mm), *c* represents pHLIP concentration (5 μM) and *N* represents the number of peptide bonds (number of amino acid residues minus one, that is, 35 in this case). All the spectra were adjusted by subtracting their own values at 260 nm (usually close to 0), and smoothed by averaging three adjacent points before plotting. The control spectrum (taken with liposome samples without pHLIP) was subtracted for each pH sample.

### Solid-state NMR spectroscopy

All the NMR measurements were performed on a Bruker 600 MHz solid-state NMR spectrometer (Bruker Corporation, Billerica, MA) installed with a 2.5 mm TriGamma MAS probe (Bruker Corporation, Bellerica, MA). The static and MAS ^31^P spectra were recorded using direct polarization radiofrequency pulse at ∼50 kHz with ∼100 kHz ^1^H continuous-wave (CW) decoupling. The spin–spin (T_2_) relaxation measurements were done using Hahn spin echo pulse sequence, which included ∼50 kHz ^31^P radiofrequency pulses and ∼100 kHz ^1^H CW decoupling. All ^31^P spectra were taken at ambient temperature.

Two-dimensional (2D) ^13^C–^13^C correlation spectra were recorded with 10 ms radiofrequency-assisted diffusion (RAD) mixing periods for the detection of intra-residue cross-peaks^53^. The RAD pulse sequence also contained ∼60 kHz ^1^H cross polarization (CP) block and 40–56 kHz ^13^C linearly ramped CP over 1.5 ms and ∼100 kHz two-pulse phase modulation ^1^H decoupling^54^. All the 2D experiments were performed with 10 kHz MAS frequency. Samples were cooled with 270 K N_2_ flow during the experiments and the sample temperature was kept at ∼280 K based on the measurements on ^1^H chemical shift in H_2_O. This temperature allowed the detection of relatively strong ^13^C signal and in the meanwhile kept the POPC liposome in the liquid crystalline phase (that is, the phase transition temperature for POPC is ∼271 K).

The one-dimensional ^13^C–^31^P *fs*REDOR spectra were recorded with the same CP amplitude as for the RAD and 45 kHz π pulses on ^13^C and/or ^31^P channels during the dephasing periods and ∼100 kHz two-pulse phase modulation ^1^H decoupling field during the acquisition. The ^13^C and ^31^P Gaussian selective pulses were set to 6.0 ms to cover the frequency range of ∼600 Hz, which corresponded to ∼4.0 p.p.m. range on ^13^C. The ^13^C transmitter was centred at 18.0 p.p.m. based on the Ala ^13^Cβ chemical shifts. The ^31^P transmitter was centred on the lipid phosphate peak. The parameters for PSL acquisition block were set as follows: 14 data points per data-collecting period (*τ*_1_), 45.0 μs dead time period (*τ*_2_), 33 kHz π pulse in the middle of one data-collecting cycle, 72 data-collecting cycles and 484 data points after the PSL train^48^. The recycle delay was set to 3 s to avoid overheating because of the long acquisition time. For *fs*REFOR experiments, the MAS frequency was set to 5 kHz and the samples were protected using 270 K cooling N_2_ flow. The actual sample temperature was ∼275 K. The typical experimental time was between 20 and 40 h for one 2D RAD spectrum and ∼48 h for a set of REDOR data with four time points.

Quantitative analysis of the ^13^C–^31^P *fs*REDOR experiments was performed by integrating the *S*_0_ and *S*_1_ peaks. The normalized REDOR dephasing was calculated from the integration values as (*S*_0_−*S*_1_)/*S*_0_. The error bars shown in Fig. 6 represented one s.d. and were determined from the spectral noise (Supplementary Equation 4). Theoretical REDOR dephasing curves were generated using SIMPSON program with different multiple-spin ^13^C–^31^P systems that mimic either the surface-bound or the membrane-inserted scenarios^55^. For the samples at pH 6.4, the experimental REDOR dephasing data was corrected to only include the contribution from the state II′. The detailed data fitting protocols were provided in Supplementary Information.

The ^13^C–^2^H REDOR experiments were performed with 45 kHz and 33 kHz π pulses on ^13^C and ^2^H channels, respectively. The magic angle spinning frequency was set to 10 kHz and the REDOR spectra were collected at 20 ms dephasing period.

## Additional information

**How to cite this article**: Shu, N. S. *et al*. Residue-specific structures and membrane locations of pH-low insertion peptide by solid-state nuclear magnetic resonance. *Nat. Commun.* 6:7787 doi: 10.1038/ncomms8787 (2015).

## Supplementary Material

Supplementary Information Supplementary Figures 1-3, Supplementary Methods and Supplementary References

## Figures and Tables

**Table 1:** Chemical shifts of Cα, Cβ and C′ for different pHLIP states* †.

	**pH 5.3 State III (p.p.m.)**			**pH 6.4 State II′ & III (p.p.m.)**			**pH 7.4 State II (p.p.m.)**		
	**Cα**	**Cβ**	**C'**	**Cα**	**Cβ**	**C′**	**Cα**	**Cβ**	**C′**
A10	52.5	15.8	177.9	52.2 (49.8)‡	16.1 (20.0)	177.8 (175.5)	50.2	20.1	175.3
A13	53.8	15.7	178.2	54.0 (52.0)	16.7 (17.8)	178.0 (175.3)	52.0	18.2	175.0
L21	56.1	38.8	176.5	56.1	39.8	176.5	NA§	NA	NA
L22	55.0	38.5	177.0	55.2	38.6	177.1	NA	NA	NA
L26	55.8	38.1	177.0	56.7	40.0	176.9	NA	NA	NA
A27	54.0	17.0	178.5	53.8	16.3	178.4	NA	NA	174.1

NA, not available.

^*^For comparison, the Cα, Cβ and C′ chemical shifts in random coil conformation are 50.8, 17.4 and 176.1 p.p.m. for Ala, and 53.4, 40.7 and 175.9 p.p.m. for Leu, respectively.

^†^All chemical shift values have ∼ 0.5 p.p.m. uncertainty judged by the experimental line-widths.

^‡^The values in parentheses represented a second set of peaks for the labelled site.

^§^The cross peak intensities were too low compared with noise.
