# Supplementary material for: Residue-specific structures and membrane locations of pH-low insertion peptide by solid-state nuclear magnetic resonance
Source: Nat Commun. 2015 Jul 21;6:7787. doi: 10.1038/ncomms8787 (PMC4518304; doi:10.1038/ncomms8787)
Supplement: Supplementary Information — Supplementary Figures 1-3, Supplementary Methods and Supplementary References [file ncomms8787-s1.pdf]

Supplementary Figure 1

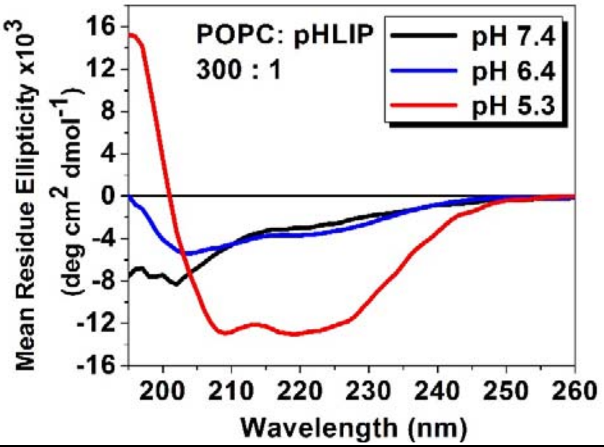

Supplementary Figure 1 CD spectra of pHLIP / POPC samples with a P/L molar ratio of 1:300. The sample preparation protocols and instrumentation parameters are provided in the Methods section of the main text. CD spectra at 1:300 P/L ratio appear very similar to those at 1:75 P/L ratio (*c.f.* Figure 1A), which confirms that the step-wise protocol used for NMR sample preparation is biophysically relevant.

Supplementary Figure 2

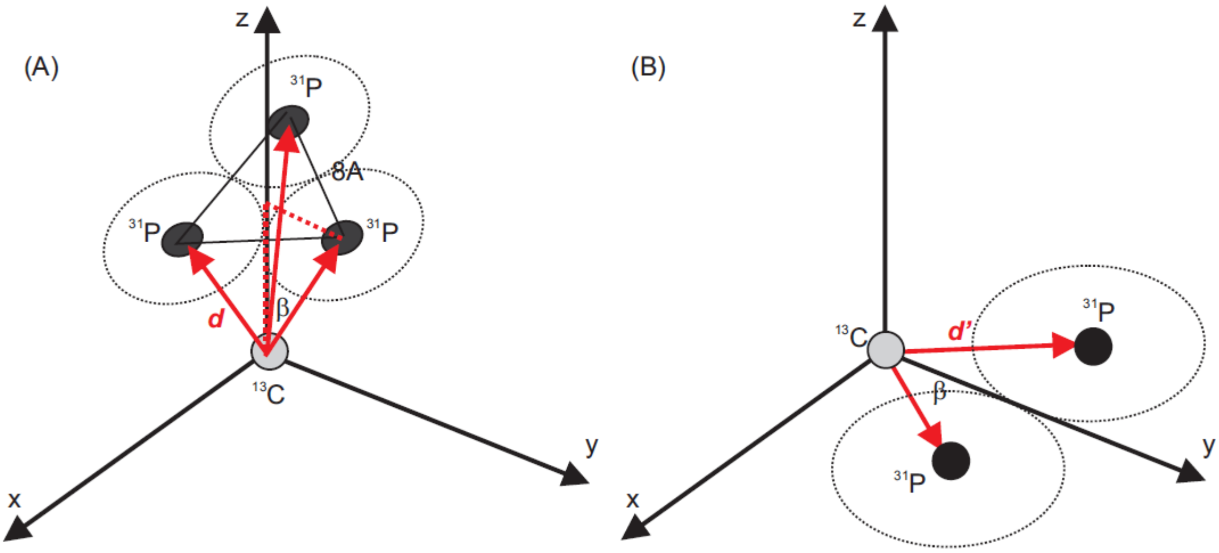

Supplementary Figure 2 Geometry of the model systems used for the SIMPSON simulations for (A) membrane-adsorbed or embedded (state II / II') and (B) membrane-inserted (state III) pHLIP peptides. The red arrows indicate individual ^{13}C-^{31}P vectors. In panel (A), the three ^{31}P nuclei are in a plane parallel to *x-y*. In panel (B), all three nuclei are in the *x-y* plane.

Supplementary Figure 3

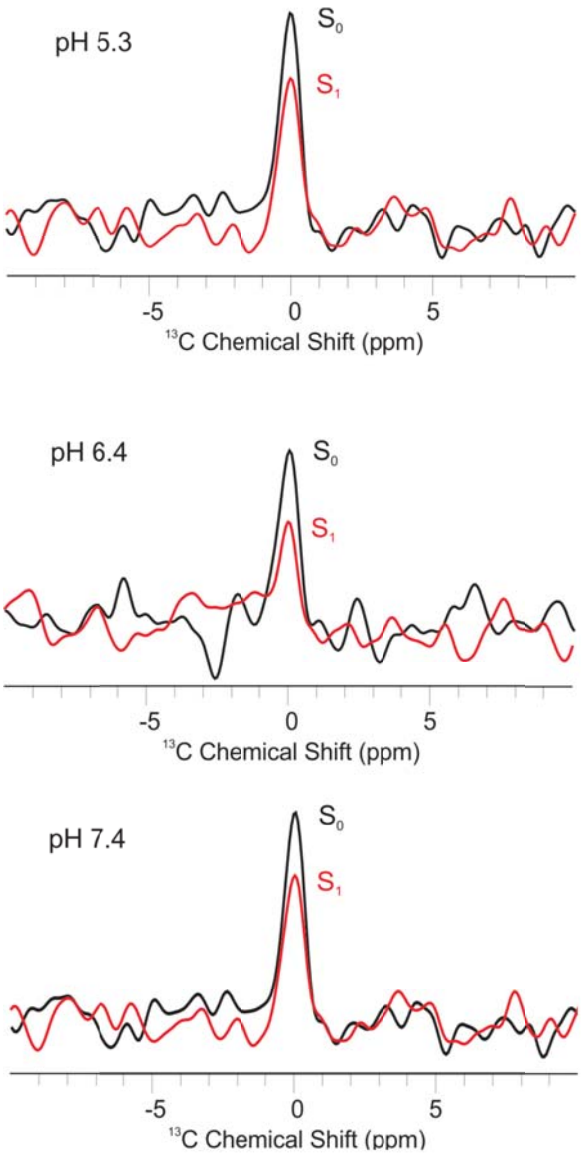

Supplementary Figure 3 ^{13}C-^{2}H REDOR S_0 (black) and S_1 (red) spectra for the Cβ of A13 in samples prepared at pH 5.3 (top), 6.4 (middle) and 7.4 (bottom) with 15% deuterated POPC. In all cases, ~30% of dephasing ($\Delta S/S_0$) was detected, which indicate close contact between the labeled ^{13}C site and ^{2}H on lipid alkyl chains.

Supplementary Methods

We utilized two different ^{13}C-^{31}P multi-spin systems to mimic the scenarios of surface-bound and membrane-inserted pHLIP molecules. For the membrane adsorbed or embedded model (Supplementary Figure 2A, representing state II/II'), we considered a 4-spin system consisting of one ^{13}C nucleus and three ^{31}P nuclei. The geometries of ^{31}P nuclei mimic the densely packed POPC phosphate headgroups with ~ 8 Å distance between the adjacent ^{31}P. In the Cartesian coordinate system shown in Supplementary Figure 2A, one of the ^{13}C-^{31}P internuclear vector is placed within the *y-z* plane while the ^{13}C nucleus is fixed at the origin point. Using this setup, the sets of Euler angles (α, β, γ) that connect the principle axis systems and molecular frames are (0, β, 0), (120, β, 0) and (240, β, 0) for vectors ^{13}C-$^{31}P_1$, ^{13}C-$^{31}P_2$, and ^{13}C-$^{31}P_3$ respectively, where β was calculated using:

$$\beta = sin^{-1}\left[4.619/\sqrt[3]{12250/d}\right] \quad (1)$$

In Eq.1, the parameter *d* represents the internuclear distance between ^{13}C and ^{31}P in the unit of Å. SIMPSON package[1] was utilized to generate simulated curves shown in Figure 6. The best-fit distances reported in the main text for samples at pH 7.4 (state II) and 6.4 (state II') represent the proximity between an individual ^{13}C nucleus to the ^{31}P plane (i.e. $h^2 = d^2 - 21.33$).

For the membrane-inserted state III, we considered a three-spin model with one ^{13}C and two ^{31}P nuclei (Supplementary Figure 2B). Additonal ^{31}P nuclei are neglected because for a TM helix there are only likely to be two annular lipids that are within REDOR distance of any sidechain Cβ. In this simple spin system, the ^{13}C is placed at the origin of the Cartesian coordinate and the plane formed by all three spins are fixed in the *x-y* plane. Therefore, the set of

Euler angles for such a system are (0, 0, 0) and (0, β, 0) for vectors ^{13}C-$^{31}P_1$ and ^{13}C-$^{31}P_2$, where β was calculated using:

$$\beta = 2sin^{-1}\left[4/\sqrt[3]{12250/d'}\right] \quad (2)$$

with the same definition for *d'* as *d* in Eq. 1. Thus, the best-fit distances reported in the main text for samples at pH 5.3 indicate the proximity between a specific $^{13}C\beta$ to the vector that connects the two ^{31}P nuclei (i.e. $h^2 = d'^2 - 16$)

For pH 6.4 samples, since there are two populations (i.e. ~ 70% unstructured state II' and ~ 30% α-helical state III, see discussion in main text), we assume that the state III population at pH 6.4 adopt the same $^{13}C\beta$-^{31}P proximity as the inserted state III at pH 5.3. Therefore, the pH 6.4 experimental data was first corrected for removing the contribution from state III using:

$$(\frac{\Delta S}{S_0})_{II'}^{corr} = \frac{1.00}{0.70}(\frac{\Delta S}{S_0})_{6.4}^{exp} - \frac{0.30}{0.70}(\frac{\Delta S}{S_0})_{5.3}^{exp} \quad (3)$$

The terms $(\frac{\Delta S}{S_0})_{6.4}^{exp}$ and $(\frac{\Delta S}{S_0})_{5.3}^{exp}$ represent the experimental REDOR dephasing at pH 6.4 and pH 5.3 for the same labeled sites, respectively.

For each REDOR dephasing value (ΔS/S0) shown in Fig. 6, the uncertainty (error bar) was calculated using the following Eq. 4:

$$\sigma^{exp} = \sqrt{S_0^2\sigma_{s1}^2 + S_1^2\sigma_{s0}^2}/S_0^2 \quad (4)$$

, where σ_{s0}^2 and σ_{s1}^2 were the experimental root-mean-square deviations of integrated intensities over 1 ppm chemical shift ranges in the spectral regions without signal.

Supplementary References

1. Bak M, Rasmussen, J.T., and Nielsen, N.C. SIMPSON: a general simulation program for solid-state NMR spectroscopy. *J Magn Reson* **147**, 296-300 (2000).
